# Comparative Outcomes of Surgical, Endovascular, Radiosurgical, and Multimodal Management of Cerebral Arteriovenous Malformations: A Systematic Review

**DOI:** 10.7759/cureus.113715

**Published:** 2026-07-31

**Authors:** Flora Alrumaih, Abdulrahman M Alharbi, Fadwa M Albattah, Deem A Almethen, Dhay S Alharbi, Faris A Alalwan, Bader S Alharbi, Raghad S Albiyebi, Fisal T Alrogibah, Ohud T Alharbi, Muath I Alfallaj

**Affiliations:** 1 Department of Surgery, College of Medicine, Qassim University, Buraydah, SAU; 2 Department of Neurosurgery, National Neuroscience Institute, King Fahad Medical City, Riyadh, SAU

**Keywords:** brain arteriovenous malformation (bavm), cerebral arteriovenous malformation, endovascular embolization, hybrid neurovascular surgery, microsurgical resection, multimodal treatment, neurological outcomes, obliteration rate, stereotactic radiosurgery, treatment complications

## Abstract

Cerebral arteriovenous malformations (AVMs) pose significant therapeutic challenges, with multiple modalities offering varying degrees of cure, risk, and functional preservation. Although microsurgery, endovascular embolization, stereotactic radiosurgery, and multimodal approaches are widely used, comparative outcomes remain heterogeneous. This systematic review synthesizes contemporary evidence across treatment strategies to guide patient-specific decision-making.

Following Preferred Reporting Items for Systematic Reviews and Meta-Analyses 2020 guidelines and a registered International Prospective Register of Systematic Reviews protocol (CRD420251066493), PubMed, MyEBSCO, and Embase were searched to June 1, 2025. Eligible studies included randomized trials, cohort studies, and case series (≥10 patients) reporting clinical outcomes after AVM treatment. Two reviewers independently performed study selection, data extraction, and risk-of-bias assessment (Risk of Bias 2, Newcastle-Ottawa Scale, National Institutes of Health tool). Qualitative synthesis was conducted due to substantial heterogeneity.

A total of 135 studies involving 27,541 patients were included. Microsurgery achieved 78%-100% obliteration, with complication rates 6.8%-60% and mortality 0%-13.3%. Embolization demonstrated variable obliteration (13%-92%) and complications (14%-25%), with mortality (0%-2%), supporting its role primarily as an adjunct. Multimodal strategies yielded 85%-100% obliteration, 7%-36% complications, and 0%-5% mortality, with hybrid workflows showing the most favorable balance. Radiosurgery produced 22%-100% obliteration, excellent functional outcomes (modified Rankin Scale 0-2 in 80%-95%), and the lowest contemporary mortality (0%-1%).

Optimal AVM management depends on individualized selection and modality sequencing rather than a single superior technique. Microsurgery provides immediate cure in suitable cases, radiosurgery offers durable long-term outcomes, and embolization enhances both when applied judiciously. Multimodal and hybrid approaches combine these strengths, achieving high cure rates with acceptable morbidity.

## Introduction and background

Brain arteriovenous malformations (bAVMs) are rare congenital vascular anomalies characterized by tangles of dilated arteries and veins lacking a normal capillary bed, which predispose patients to hemorrhage, neurological deficits, and even death [1]. Although often asymptomatic, their first presentation can be catastrophic. Due to their rarity and frequent subclinical course, the exact incidence remains uncertain, but estimates suggest a prevalence of 1.3 cases per 100,000 person-years [2]. Complete eradication of the nidus is the primary treatment goal to prevent hemorrhagic events and improve outcomes.

Current therapeutic options for bAVMs include microsurgical excision, endovascular embolization, stereotactic radiosurgery (SRS), or a combination of these modalities [1]. Treatment decisions are often guided by the Spetzler-Martin (S-M) grading system, introduced in 1986 and revised in 2011, which stratifies arteriovenous malformations (AVMs) based on size, venous drainage, and eloquence of adjacent brain tissue. Microsurgery is typically favored for low-grade lesions (S-M I-II), while S-M III lesions may require multimodal strategies. Higher grade lesions (S-M IV-V) are often approached conservatively or with radiosurgery and/or embolization, depending on individual risk-benefit profiles. Embolization is frequently used as an adjunct to reduce nidus size, facilitate surgery or radiosurgery, or, in select cases, achieve complete obliteration with agents such as Onyx (Medtronic, Minneapolis, MN) or coils [3].

Recent advancements in imaging, embolic materials, and surgical techniques have improved treatment safety and efficacy. However, management of unruptured AVMs remains controversial, especially following the A Randomised trial of Unruptured Brain Arteriovenous malformations (ARUBA) trial, which reported worse outcomes with intervention compared to medical management. These findings, though influential, have been challenged due to limitations in methodology, short follow-up, and narrow generalizability. Clinical practice continues to vary widely across centers, particularly in the use of multimodal therapy and selection criteria for surgery, highlighting the need for high-quality, comparative outcome data [2].

While many studies have evaluated individual treatment modalities, few have systematically compared their long-term outcomes or examined multimodal strategies in a standardized fashion. Moreover, functional outcomes, such as neurological status and quality of life, are often underreported in favor of angiographic obliteration or hemorrhage rates. There is a pressing need for updated and comprehensive synthesis of the available evidence to guide clinical decision-making and refine treatment algorithms.

This systematic review aims to evaluate and compare the clinical outcomes of surgical, endovascular, radiosurgical, and multimodal treatment strategies for cerebral AVMs. Key outcomes of interest include nidus obliteration, hemorrhage rates, neurological status, complications, and mortality. To ensure full transparency, we note that this work has not been previously presented or disseminated in any form, including abstracts, posters, or oral presentations.

## Review

Materials and methods

Study Design and Reporting Standards

This systematic review was conducted in accordance with the Preferred Reporting Items for Systematic Reviews and Meta-Analyses (PRISMA) 2020 guidelines [4]. The protocol was prospectively registered in the International Prospective Register of Systematic Reviews (PROSPERO; Registration ID: CRD420251066493) [5].

Eligibility Criteria

Studies were eligible for inclusion if they reported clinical outcomes of cerebral AVMs treated with surgical, endovascular, radiosurgical, or multimodal approaches. All patient populations were considered, including individuals of any age with either ruptured or unruptured AVMs. Eligible study designs consisted of randomized controlled trials, cohort studies, and case series that enrolled at least 10 patients. To be included, studies were required to report at least one clinically relevant outcome, such as obliteration rate, hemorrhage, neurological outcomes, complications, or mortality.

Studies were excluded if they were case reports, conference abstracts, narrative or systematic reviews, or animal studies. Research focusing exclusively on diagnostic techniques without reporting treatment outcomes was also excluded. In addition, studies that evaluated spinal AVMs rather than cerebral AVMs were not considered eligible.

Search Strategy

A comprehensive search strategy was developed using controlled vocabulary (Medical Subject Headings (MeSH) and Emtree terms) and free-text keywords. Searches were conducted in PubMed, Embase, and MyEBSCO and were limited to English-language studies published between January 1, 2000, and June 1, 2025. Additional eligible studies were identified by manually screening the reference lists of included articles.

PubMed: The PubMed search combined AVM-related MeSH terms and keywords (“brain arteriovenous malformation”, “cerebral arteriovenous malformation”, “cerebral AVM”, “brain AVM”) with treatment-related terms (“surgery”, “microsurgery”, “resection”, “embolization”, “endovascular”, “radiosurgery”, “stereotactic radiosurgery”, “Gamma Knife”, “treatment outcomes”) using the Boolean operator AND. The full Boolean structure was: (brain arteriovenous malformation OR cerebral arteriovenous malformation OR cerebral AVM OR brain AVM) AND (surgery OR microsurgery OR resection OR embolization OR endovascular OR radiosurgery OR stereotactic radiosurgery OR Gamma Knife OR treatment outcomes) AND (publication date: 2000-2025).

Embase: The Embase search used the Emtree term “brain arteriovenous malformation” (exploded) together with the keywords “cerebral arteriovenous malformation” and “brain AVM”. These terms were combined with treatment-related terms (“microsurgery”, “neurosurgery”, “embolization”, “endovascular procedure”, “radiosurgery”, “Gamma Knife”, “stereotactic radiosurgery”, “treatment outcome”) using AND. Publication years were limited to 2000-2025, and results were restricted to English-language articles.

MyEBSCO: The MyEBSCO search combined AVM-related keywords (“brain AVM”, “cerebral AVM”, “arteriovenous malformation”) with treatment terms (“surgery”, “microsurgical resection”, “endovascular embolization”, “radiosurgery”, “treatment outcome”, “complications”, “obliteration”) using AND. Filters limited results to English-language studies published between 2000 and 2025.

Data Extraction

Data extraction was performed independently by two reviewers using a standardized Excel form (Microsoft Corporation, Redmond, WA). Extracted information included key study characteristics such as the study title, authors, year of publication, country of origin, and study design. Patient and lesion characteristics were also recorded, including sample size, AVM size, anatomical location, and S-M grade. Treatment-related variables were documented for each study, including the specific modality applied and the embolic agent used when applicable.

Primary outcomes consisted of the reported obliteration rate and each study’s definition of cure. Secondary outcomes included hemorrhage rates, complications, mortality, types of complications, duration of follow-up, and functional outcomes, with good functional status defined as a modified Rankin Scale (mRS) score of 2 or lower. Key findings from each study were recorded in detail. Any discrepancies between reviewers were resolved through discussion and consensus.

RoB Assessment

Randomized controlled trials were evaluated using the Cochrane Risk of Bias 2.0 (RoB 2.0) tool (Cochrane, London, UK) [6]. Observational cohort and case-control studies were assessed using the Newcastle-Ottawa Scale (NOS) [7]. Case series, for which NOS is not applicable, were appraised with the National Institutes of Health (NIH) Quality Assessment Tool for Case Series Studies [8]. All assessments were performed independently by two reviewers, with disagreements resolved through discussion.

Study Selection

**Figure 1:**
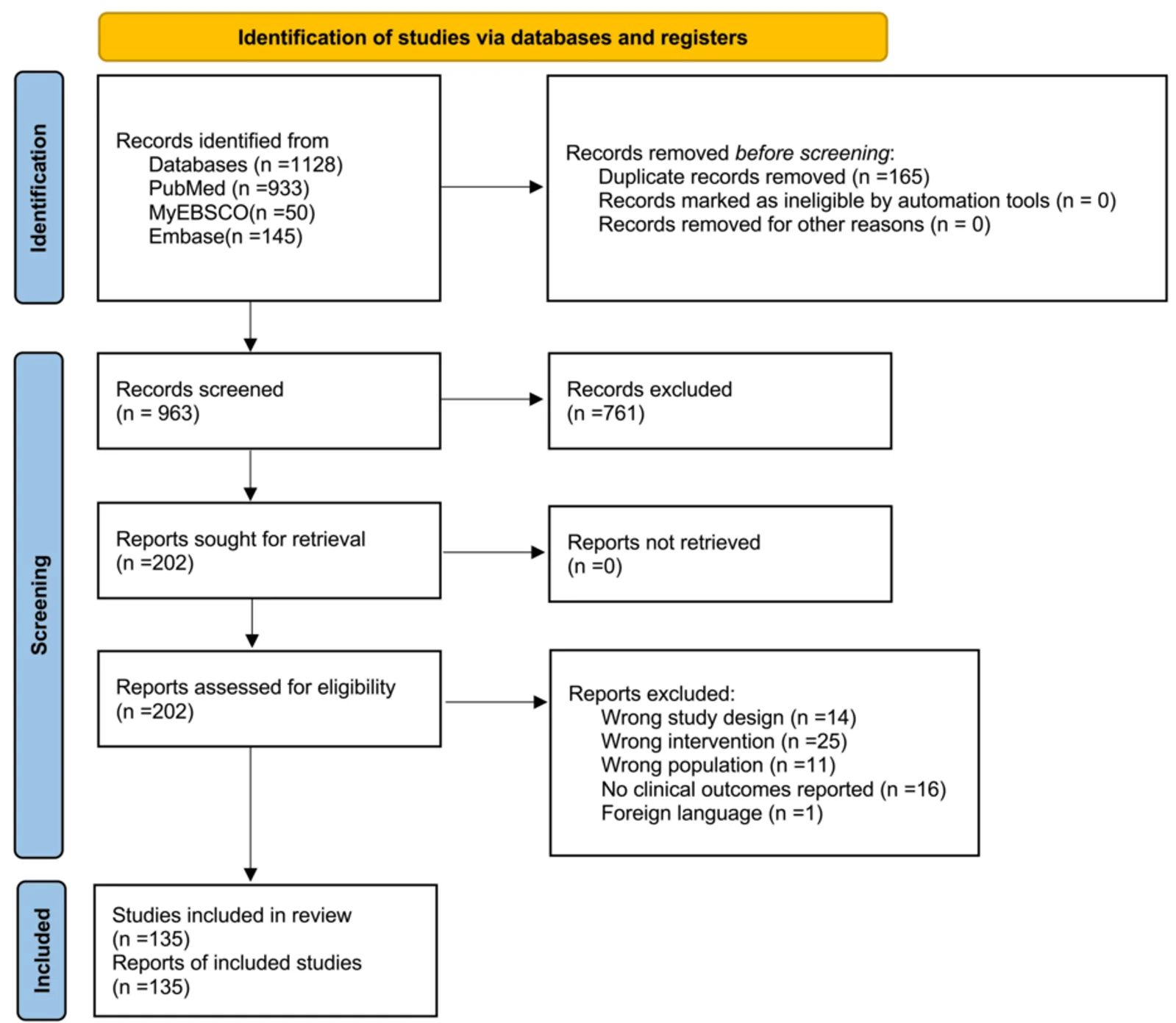
PRISMA 2020 flow diagram for study selection PRISMA: Preferred Reporting Items for Systematic reviews and Meta-Analyses

Two reviewers independently screened titles and abstracts for eligibility, followed by full-text assessment. Disagreements were resolved by consensus or, when necessary, adjudication by a third reviewer. The study selection process is detailed in the PRISMA 2020 flow diagram (Figure 1). The characteristics of the included studies, including authors, publication year, country, study design, sample size, AVM location, S-M grade, treatment modality, and follow-up duration, are summarized in Table 1.

**Table 1:** Characteristics of the included studies Values reported under “Analyzed Patients” represent the final analyzed patient populations contributing extractable outcome data within each included study. These values correspond to the populations included in outcome analyses rather than initially screened or treated populations. The cumulative total is consistent with the 27,541 patients reported in the Results section. Values represent data extracted from all included studies AVM: arteriovenous malformation; S-M: Spetzler-Martin grade; NR: not reported; SRS: stereotactic radiosurgery; HFSRT: hypofractionated stereotactic radiosurgery; LINAC: linear accelerator-based radiosurgery; NBCA: n-butyl cyanoacrylate; RCT: randomized controlled trial

Study (year)	Country	Design	Analyzed patients	AVM location	S-M grade	Treatment modality	Follow-up	
Oulasvirta et al. [9] (2023)	Finland	Cohort	41	Supratentorial	l-V	Multimodality	Median 19.1 years	
Gawish et al. [10] (2022)	Germany	Cohort	68	NR	NR	LINAC SRS	Mean 35 months	
Ozden et al. [11] (2023)	Turkey	Cohort	62	Mixed	III-IV	Multimodality	NR	
Loebel et al. [12] (2022)	Germany and Italy	Cohort	123	Mixed	I-V	Multimodality	Median 48.1 months	
Zhu et al. [13] (2024)	China	Cohort	201	Mixed	I-IV	Gamma knife SRS	NR	
Jiang et al. [1] (2022)	China	Cohort	130	Mixed	I-V	Multimodality	Mean 37.4 months	
Sato et al. [14] (2020)	Japan	Cohort	788	Mixed	I-V	Endovascular embolization	30 days	
Shah et al. [15] (2024)	USA	Cohort	37	Cortical	NR	Cyberknife SRS	Mean 26.2 months	
Walcott et al. [16] (2014)	USA	Case series	44	Mixed	NR	Proton beam SRS	Median 52 months	
Baharvahdat et al. [17] (2014)	France	Cohort	408	Mixed	l-V	Endovascular embolization	Short-term only (≤1 month; no long-term duration reported)	
								van Essen et al. [18] (2018)	The Netherlands	Cohort	25	Mixed	I-V	Multimodality	Mean 11.5 years	
Brauner et al. [19] (2025)	France	Case series	46	Mixed	I-V	Multimodality	Median 6 months	
Potts et al. [20] (2015)	USA	Cohort	232	Mixed	I-II	Surgery	Mean 1.7 years	
Jordan et al. [21] (2014)	Cuba	Cohort	71	Mixed	l-V	Endovascular embolization	Mean 31.1 months	
Baharvahdat et al. [22] (2019)	France	Cohort	224	Mixed	I-II	Endovascular embolization	Mean 9.7 months	
Lv et al. [23] (2010)	China	Cohort	30	Supratentorial	NR	Endovascular embolization	Mean 80 months	
Kim et al. [24] (2019)	South Korea	Cohort	264	Mixed	I-IV	Gamma knife SRS	Mean 55.5 months	
Shekhtman et al. [25] (2015)	Russia	Cohort	93	Mixed	I-V	Multimodality	Mean 27.6 months	
Hellstern et al. [26] (2022)	Germany	Case series	47	NR	I-V	Endovascular embolization	NR	
Hancevic et al. [27] (2025)	Croatia	Cohort	241	Mixed	l-V	Gamma knife SRS	Mean 3 years	
Gao et al. [28] (2022)	China	Cohort	88	Mixed	I-V	Gamma knife SRS	Median 65 months	
Rutledge et al. [29] (2014)	USA	Cohort	74	Mixed	I-V	Multimodality	Mean 21 months	
Pierot et al. [30] (2013)	France	Case series	20	Supratentorial	I-IV	Multimodality	Median 3.5 years	
Smrcka et al. [3] (2021)	Czech Republic	Case series	50	Mixed	I-IV	Surgery	3 months	
Abla et al. [31] (2015)	USA	Case series	16	Supratentorial	lll-V	Multimodality	Mean 6.9 years	
Sanchez-Mejia et al. [32] (2009)	USA	Cohort	42	NR	I-IV	Multimodality	Mean 7.1 years	
Arslan et al. [33] (2017)	Turkey	Cohort	199	Mixed	I-V	Gamma knife SRS	Median 60 months	
Lee et al. [34] (2024)	United Kingdom	Cohort	88	Mixed	I-III	Multimodality	NR	
van Rooij et al. [35] (2012)	The Netherlands	Case series	23	Mixed	NR	Endovascular embolization	Mean 21 months	
van Rooij et al. [36] (2012)	The Netherlands	Case series	24	Supratentorial	I-III	Curative embolization	3 months	
Josephson et al. [37] (2012)	United Kingdom	Cohort	229	Supratentorial	NR	Multimodality	Median 9 years	
Tos et al. [38] (2025)	North America and Europe	Cohort	96	Mixed	IV	Multimodality	Median 24 months	
Pan et al. [39] (2009)	China	Case series	20	Supratentorial	l-lV	Multimodality	3-24 months	
Chen et al. [40] (2023)	China	Cohort	622	NR	I-V	Endovascular embolization	Median 5.5 years	
Maryashev et al. [41] (2015)	Russia	Case series	315	Mixed	II-V	SRS	Median 2.5 years	
He et al. [42] (2018)	China	Cohort	10	Supratentorial	I-IV	Transvenous embolization	Median 8 months	
Mohr et al. [43] (2017)	Multinational	RCT	223	NR	I-II	Comparative multimodality study	Median 33.3 months	
Chen et al. [44] (2021)	China	Cohort	71	Supratentorial	I-V	Comparative multimodality study	Mean 4.2 years	
Liu et al. [45] (2010)	China	Case series	126	Supratentorial	NR	Onyx embolization	Median 33 months	
Tos et al. [46] (2025)	Multicenter (USA and Europe)	Cohort	180	Mixed	I-II	Comparative multimodality study	Median 12-36 months	
Branko et al. [47] (2025)	Italy	Cohort	209	Mixed	I-V	Multimodality	Median 54 months	
Mounayer et al. [48] (2007)	France	Case series	94	Mixed	I-V	Onyx embolization	Median 4.5 months	
Patel et al. [49] (2008)	India	Case series	54	Mixed	I-V	LINAC SRS	Median 33 months	
Karlsson et al. [50] (2022)	Multinational	Cohort	5,037	NR	NR	Gamma knife SRS	2 years	
Alexander et al. [51] (2018)	USA	Case control	160	NR	NR	Comparative multimodality study	Median 1.6 years	
Nakai et al. [52] (2012)	Japan	Case series	19	NR	I-V	Multimodality	Mean 39.2 months	
Panagiotopoulos et al. [53] (2009)	Germany	Case series	82	Mixed	I-V	Onyx embolization	Mean 8.8 months	
Huang et al. [54] (2024)	Taiwan	Cohort	262	Mixed	I-V	SRS	Median 61.8 months	
Sheth et al. [55] (2014)	USA	Cohort	42	Mixed	II-V	Gamma knife SRS	3 years	
Kondo et al. [56] (2014)	Japan	Cohort	987	Mixed	I-V	Endovascular embolization	30 days	
Weber et al. [57] (2007)	Germany	Case series	93	Mixed	I-V	Onyx embolization	Mean 9.5 months	
Jayaraman et al. [58] (2008)	USA	Cohort	192	Deep	I-V	Endovascular embolization	NR	
Chye et al. [59] (2020)	Taiwan	Cohort	1,515	NR	NR	Gamma Knife SRS	15 years	
Han et al. [60] (2023)	China	Cohort	1,348	NR	I-III	Surgery and SRS	Mean 6.4 years	
Galaktionov et al. [61] (2017)	Russia	Cohort	40	Mixed	I-V	Multimodality	NR	
Javalkar et al. [62] (2009)	USA	Case series	37	Mixed	II-V	Gamma knife SRS	Median 23 months	
Ahmetspahic et al. [63] (2021)	Japan	Case series	11	Mixed	I-V	Multimodality	Median 39.5 months	
Jordan et al. [64] (2014)	Cuba	Cohort	71	Mixed	I-V	Endovascular embolization	Mean 31.1 months	
Chowdhury et al. [65] (2015)	Bangladesh	Cohort	60	Mixed	NR	Comparative multimodality study	1.3 years	
Peciu-Florianu et al. [66] (2020)	France	Cohort	172	Mixed	I-IV	Gamma knife radiosurgery	Median 8.8 years	
Punyawai et al. [67] (2021)	Thailand	Cohort	166	Mixed	NR	CyberKnife SRS	Median 72.45 months	
Iosif et al. [68] (2022)	France	Case series	22	Mixed	I-V	Transvenous embolization	12 months	
Huo et al. [69] (2015)	China	Cohort	86	Mixed	I-V	Multimodality	Mean 42 months	
Heidenreich et al. [70] (2006)	Germany	Cohort	66	NR	NR	Endovascular embolization	NR	
Feng et al. [71] (2017)	China	Case series	52	Mixed	I-IV	Surgery	Mean 22.5 months	
Maruyama et al. [72] (2005)	Japan	Cohort	500	NR	III	Gamma knife SRS	Median 7.8 years	
Morgan et al. [73] (2015)	Australia	Cohort	112	Supratentorial	III	Surgery	Median 3 years	
Hauck et al. [74] (2009)	USA	Cohort	41	Supratentorial	I-V	Onyx embolization	NR	
Sousa et al. [75] (2016)	Brazil	Cohort	90	Mixed	III	Multimodality	3 years	
Esteves et al. [76] (2008)	Brazil	Case series	61	Mixed	III-IV	Radiosurgery	Mean 28.8 months	
Abla et al. [77] (2015)	USA	Cohort	174	Supratentorial	I-V	Surgery	Median 5.3 years	
Abud et al. [78] (2011)	Brazil and France	Case series	17	Mixed	I-IV	Onyx embolization	6 months	
Dănăilă [79] (2012)	Romania	Case series	46	Mixed	I-IV	Surgery	6 months	
de los Reyes et al. [80] (2011)	USA	Cohort	43	Mixed	I-V	Onyx embolization	Median 27.5 months	
								He et al. [81] (2019)	China	Cohort	21	Mixed	I-IV	Transvenous embolization	Median 5.5 months	
De Sousa et al. [82] (2020)	France	Cohort	57	Mixed	I-V	Endovascular embolization	6 months	
Potts et al. [83] (2014)	USA	Cohort	215	Mixed	II-V	SRS	3 years	
Gao et al. [84] (2014)	China	Case series	22	Mixed	I-IV	Endovascular embolization	Mean 15 months	
Raupp and Fernandes [85] (2005)	Brazil	Cohort	104	Mixed	NR	NBCA embolization	8 years	
Komatsu et al. [86] (2020)	Japan	Cohort	242	NR	I-V	Transvenous embolization	NR	
Copelan et al. [87] (2020)	USA	Cohort	115	Mixed	NR	Surgery	Mean 2.3 years	
Cellerini et al. [88] (2002)	Italy	Case series	10	Mixed	NR	Multimodality	Mean 23 months	
Takagi et al. [89] (2012)	Japan	Case series	38	NR	III-V	Surgery	NR	
Reynolds et al. [90] (2019)	USA	Cohort	NR	NR	NR	Comparative multimodality study	NR	
Blamek et al. [91] (2013)	Poland	Case series	10	Supratentorial	II-IV	SRS	Median 38.5 months	
Mukherjee et al. [92] (2017)	India	Case series	14	Mixed	II-V	Gamma knife SRS	Median 35.6 months	
								Starke et al. [93] (2009)	USA	Cohort	202	NR	I-IV	Multimodality	Mean 43.4 months	
Oermann et al. [94] (2016)	USA	Cohort	1,810	Mixed	NR	SRS	Median 3-4 years	
Jalali et al. [95] (2009)	India	Case series	23	Mixed	II-III	SRS	Median 22 months	
Yu et al. [96] (2004)	China	Case series	27	Mixed	I-V	Endovascular embolization	2-7 years	
Stapf [97] (2010)	Multinational	RCT	800	NR	NR	Comparative multimodality study	Planned ≥5 years	
Lang et al. [98] (2012)	USA	Cohort	28	Supratentorial	I-IV	Surgery	Median 15 months	
Sirakov et al. [99] (2020)	Bulgaria	Case series	16	NR	I-IV	Endovascular embolization	Mean 5.1 months	
Mohan et al. [100] (2024)	India	Case series	15	Supratentorial	III	LINAC SRS	Median 15 months	
Qureshi et al. [101] (2020)	Multinational	Subgroup analysis of RCT	26	NR	I-IV	Endovascular embolization	Median 11.8 months	
Liu et al. [102] (2014)	China	Case series	31	Mixed	I-II	Curative embolization	3-6 months	
Kim et al. [103] (2020)	South Korea	Cohort	98	NR	I-IV	Multimodality	Median 47 months	
Chen et al. [104] (2021)	Multinational	Cohort	1,178	Mixed	NR	Multimodality	Mean 4-6 years	
Arai et al. [105] (2006)	Japan	Cohort	13	Supratentorial	II-IV	Multimodality	Mean 51.6 months	
Sobh and Hegazy [106] (2013)	Egypt	Case series	15	Mixed	I-V	Endovascular embolization	3 months	
Tomsick et al. [107] (2002)	USA	RCT	104	NR	I-V	Endovascular embolization	NR	
Sun et al. [108] (2025)	Taiwan	Cohort	75	Mixed	II-III	Gamma knife SRS	Median 104 months	
Bitaraf et al. [109] (2017)	Iran	Cohort	388	Mixed	I-V	Gamma knife SRS	Mean 61.6 months	
Yan et al. [110] (2021)	China	Cohort	152	Mixed	I-II	Multimodality	Mean 6.2 years	
Blamek et al. [111] (2013)	Poland	Cohort	49	Mixed	II-V	HFSRT	Median 23.8 months	
Toader et al. [112] (2024)	Romania	Cohort	128	Mixed	I-V	Multimodality	2 years	
Shuto and Matsunaga [113] (2021)	Japan	Cohort	19	Mixed	III-V	Gamma knife SRS	Mean 3.9 years	
Leonardi et al. [114] (2005)	Italy	Case series	34	Mixed	III-V	Multimodality	NR	
Choe et al. [115] (2008)	South Korea	Case series	100	Mixed	I-V	Gamma knife SRS	Mean 37.5 months	
Lee et al. [116] (2009)	South Korea	Cohort	23	Mixed	II-V	Gamma knife SRS	Mean 41.2 months	
Conger et al. [117] (2016)	USA	Cohort	11	Mixed	I-IV	Multimodality	Median 2 months	
Pierot et al. [118] (2005)	France	Cohort	48	Mixed	NR	Endovascular embolization	NR	
Winkler et al. [119] (2020)	USA	Cohort	189	NR	I-V	Multimodality	Mean 4.1 years	
Sethi et al. [120] (2023)	USA	Case series	16	Mixed	II-IV	SRS	Median 35 months	
Koo et al. [121] (2024)	Korea	Cohort	154	Mixed	I-V	Gamma knife SRS	Mean 52.1 months	
Fang et al. [122] (2022)	China	Case series	150	Mixed	I-V	Multimodality	30 days	
Miyachi et al. [123] (2017)	Japan	Cohort	73	Mixed	I-V	Multimodality	3 years	
Raymond et al. [124] (2005)	Canada	Cohort	227	Mixed	NR	Multimodality	Mean 32 months	
Elewa [125] (2018)	Egypt	Case series	21	Supratentorial	II	Endovascular embolization	Mean 45.5 months	
Nagashima et al. [126] (2004)	Japan	Cohort	126	Mixed	I-V	Multimodality	NR	
Sugiu et al. [127] (2004)	Japan	Cohort	68	Mixed	I-IV	Endovascular embolization	NR	
Wang et al. [128] (2021)	China	Cohort	38	Mixed	IV-V	Multimodality	Median 4.5 months	
He et al. [129] (2022)	China	Cohort	27	Supratentorial	I-IV	Endovascular embolization	Median 36 months	
Zeng et al. [130] (2022)	China	Cohort	132	Mixed	I-IV	Multimodality	NR	
Maalim et al. [131] (2023)	China	Cohort	169	Mixed	I-V	Surgery	Mean 28 months	
Miron et al. [132] (2024)	Romania	Cohort	191	Mixed	I-V	Surgery	Mean 28 months	
Li et al. [133] (2005)	China	Cohort	469	NR	I-V	Endovascular embolization	NR	
Behzadi et al. [134] (2022)	USA	Cohort	30	Mixed	I-V	Endovascular embolization	Mean 39 months	
Hashim et al. [135] (2016)	Malaysia	Cohort	13	Mixed	I-IV	Endovascular embolization	NR	
Song et al. [136] (2005)	China	Case series	50	Mixed	NR	Onyx embolization	6 months	
Bai et al. [137] (2019)	China	Case series	11	Supratentorial	I-III	Endovascular embolization	Mean 17 months	
Hong et al. [138] (2024)	South Korea	Cohort	55	Mixed	I-II	Surgery	90 days	
Yu et al. [139] (2025)	China	Cohort	213	Mixed	I-V	Multimodality	Mean 49.9 months	
De Witt et al. [140] (2021)	USA	Cohort	956	NR	NR	Gamma knife SRS	30 days	
Morgan et al. [141] (2000)	Australia	Cohort	250	Mixed	I-V	Multimodality	12 months	

Data Synthesis

Data synthesis was performed qualitatively because the included studies demonstrated substantial clinical and methodological heterogeneity in study design, patient populations, AVM characteristics (including rupture status, S-M grade, and lesion morphology), treatment protocols, outcome definitions, and follow-up duration. These differences precluded meaningful quantitative pooling and made statistical comparisons across studies inappropriate. Accordingly, a meta-analysis was not performed. Instead, results were systematically organized according to treatment modality (microsurgical resection, endovascular therapy, SRS, and multimodality treatment) and synthesized descriptively to identify consistent trends in obliteration, hemorrhage, neurological outcomes, complications, mortality, and functional outcomes while acknowledging variation across studies.

Results

A total of 135 studies, comprising 27,541 analyzed patients, met the inclusion criteria (Figure 1). The included studies were published between 2000 and 2025 and originated from multiple countries, with China, the USA, and Japan among the most represented. Study designs consisted of four randomized controlled trials, 89 cohort studies, 41 case series, and one case-control study.

RoB Assessment

Using the NOS, a total of 89 observational studies were evaluated for methodological quality. Most studies demonstrated strong design features, with 57.8% rated as high quality and 31.1% rated as moderate quality. A smaller proportion, 7.8%, were considered fair, while 3.3% were classified as poor quality. The most common methodological limitations involved inadequate control for key confounders within the comparability domain, which reduced the internal validity of several studies. Other recurring issues included unclear definitions of the nonexposed cohort, insufficient detail regarding participant selection, and incomplete or inadequate follow-up durations, all of which limited the ability to assess long-term outcomes reliably.

Four randomized controlled trials were assessed using the Cochrane RoB 2 tool. Of these, two trials demonstrated “some concerns,” primarily due to issues related to the randomization process, deviations from intended interventions, or selective reporting. One trial met the criteria for low RoB across all domains, reflecting strong methodological rigor and transparent reporting practices.

Among the 41 case series evaluated with the NIH Quality Assessment Tool, the majority (92.9%) were rated as good quality, with the remaining 7.1% classified as fair. Despite generally adequate clinical descriptions, several studies demonstrated recurring weaknesses, such as incomplete follow-up, limited reporting of statistical methods, and nonconsecutive case enrollment, which may introduce selection bias and reduce the generalizability of findings.

In addition, the single case-control study included in this review was assessed using the NOS and was rated as high quality. This study demonstrated appropriate case and control selection, reliable exposure assessment, and adequate adjustment for potential confounders, supporting the overall robustness of its methodological design.

Results by Treatment Modality

Surgical resection: A total of 27 studies reported outcomes related to surgical resection, encompassing 1,641 surgically treated patients. Although 17 studies were categorized under the surgical subgroup in the characteristics table, additional outcome data were obtained from 20 multimodal studies that nevertheless presented discrete and separable results for their surgery-only cohorts. Of these, 13 studies evaluated microsurgical resection exclusively, while the remaining multimodal cohorts provided isolated surgical outcomes sufficiently detailed for inclusion. Together, these data form the most comprehensive synthesis of contemporary microsurgical results across diverse patient populations and AVM characteristics.

Functional outcomes varied substantially across studies, reflecting differences in case selection, surgical strategy, and AVM angioarchitecture. Favorable outcomes (mRS 0-2), whether directly reported or derived from postoperative Rankin distributions, ranged from 4.9% to 92.9% across included studies, with the highest rates observed in contemporary surgically treated low-grade AVM cohorts demonstrating high angiographic cure and favorable functional recovery [38]. Neurological deterioration, although generally infrequent, was observed in specific subgroups; in the series by Morgan et al., minor treatment-related neurological disability occurred in 4.9% of surgically treated patients who did not undergo preoperative embolization [141]. While most studies used the mRS, Cellerini et al. assessed functional outcome using the Barthel Index in all patients, reporting scores of 95-100 in most cases and 50 in one patient with significant residual deficit [88].

Obliteration outcomes were described in 17 studies and demonstrated a wide but clinically meaningful range from 78% to 100%. Complete obliteration was achieved in two microsurgical cohorts, both of which reported 100% angiographic cure following surgery [38,61]. Similarly, a single-center experience achieved complete bAVM resection in all treated patients with no mortality, underscoring the curative potential of microsurgical management in appropriately selected cases [63]. In contrast, a contemporary surgical series reported complete resection at first operation in 78% of cases treated with microsurgery alone, with incomplete resection occurring in 22% of patients [34].

Complication rates likewise demonstrated considerable variability. In an elderly AVM cohort undergoing intervention, perioperative complications occurred in 60% of patients, including intracranial hemorrhage (13.3%), intracranial infection (23.3%), new neurological deficits (20.0%), deep venous thrombosis (20.0%), major adverse cardiac events (6.7%), pulmonary infections (16.7%), and electrolyte disturbances (23.3%) [44]. Despite the established role of anatomical grading systems in estimating surgical risk, this prospective series found no significant association between individual anatomical variables, such as deep venous drainage, eloquence, or perforator supply, and adverse postoperative outcomes [73]. In a contemporary single-center microsurgical series, postoperative morbidity included motor deficit worsening (5.1%), aphasia (3.8%), postoperative hematoma (5.1%), cerebrospinal fluid leakage (2.5%), and late bone-flap infection (1.3%), illustrating the spectrum of surgery-related complications encountered in clinical practice [132].

Hemorrhagic complications were among the most frequently reported adverse events in elderly patients undergoing intervention for brain AVMs. Perioperative intracranial hemorrhage occurred in 13.3% of treated patients. Delayed postoperative hemorrhage was uncommon, with only one subsequent hemorrhagic event observed during follow-up, corresponding to an annualized rupture risk of 0.8%. New-onset neurological deficits were reported in 20.0% of patients, and long-term unfavorable neurological outcome (mRS >2) was observed in 36.7% [44].

Mortality was reported in 18 studies and ranged from 0% to 13.3%. In an elderly interventional cohort, four of 30 patients (13.3%) died, reflecting the substantial procedural and comorbidity burden in this population [44]. To maintain accuracy within the surgical subgroup, studies that did not provide mortality data specifically attributable to surgical intervention were excluded from mortality synthesis.

Endovascular treatment: A total of 41 studies reported outcomes of endovascular therapy in 4,620 patients with cerebral AVMs. Although 42 studies overall described endovascular interventions, only 41 provided analyzable outcome data and were included in this synthesis. Onyx and n-butyl cyanoacrylate (NBCA) were the most frequently used embolic agents, while Glubran, silk sutures, particles, and detachable coils were utilized less commonly. Several studies described the use of multimaterial embolization, such as combining Onyx with NBCA, or staged embolization performed across multiple treatment sessions, particularly for complex or high-flow AVMs [17,85].

In a prospective series of transvenous embolization with temporary arterial flow arrest, Iosif et al. reported good clinical outcomes (mRS < 2) in 95.5% of patients at one-year follow-up, with no procedure-related mortality [68]. In a large single-center series of 224 low-grade AVMs treated endovascularly, Baharvahdat et al. reported good functional outcomes (mRS 0-2) in 80% of patients, with 94% demonstrating stable or improved neurological status and a permanent deficit rate of 5% [17].

In a nationwide Japanese registry study of 1,042 endovascular procedures, Sato et al. reported favorable 30-day functional outcomes (mRS 0-2) in 71.3% of cases, poor outcomes (mRS 3-6) in 28.7%, and a 30-day mortality rate of 0.8% [14]. Collectively, across studies reporting functional results, favorable outcomes (mRS 0-2) ranged between 71% and 95%, confirming that most patients retained functional independence following embolization.

Obliteration outcomes varied depending on AVM grade, embolic material, and treatment strategy. In a large single-center series of low-grade AVMs, Baharvahdat et al. achieved 92% complete angiographic obliteration (205/224 patients), with 62.1% cured in a single session and only 8% demonstrating residual AVM following endovascular therapy [17]. In contrast, a 2005 Arquivos de Neuro-Psiquiatria series of NBCA embolization as primary therapy reported complete obliteration in only 5% of patients, with 36% achieving >2/3 obliteration and 49% achieving 1/3-2/3 nidus reduction [85].

For high-grade AVMs, Wang et al. reported that endovascular embolization is primarily performed to reduce nidus blood flow and facilitate subsequent microsurgical resection rather than to achieve definitive cure [128]. Overall, across the included literature, complete angiographic obliteration after embolization ranged from 5% to 92%, reflecting the heterogeneity of lesion grades, embolic agents, and procedural intent.

Complications were reported in 14%-25% of patients and reflected a mixture of hemorrhagic, ischemic, and procedure-specific events. Hemorrhagic complications have also been reported in endovascular AVM treatment. In a series by Abud et al., hemorrhagic events occurred in 11.8% of patients, including pedicle artery rupture during catheter withdrawal and nidus rupture during Onyx injection [78]. Iosif et al. reported an infarction rate of 9%, including 4.5% symptomatic and 4.5% silent infarctions, all of which were nonfatal and largely reversible [68]. In the same series, vessel perforation occurred in approximately 8%, while transient neurological deficits, either symptomatic or imaging-only, were noted in 9% of cases. These findings align with broader ranges of 6%-11% for periprocedural hemorrhage and 4%-9%for ischemic complications reported across other studies.

Residual AVMs frequently required additional treatment. Pan et al. reported that among 20 patients treated with Onyx embolization, microsurgical resection was performed in 25% of cases and SRS in 10% to achieve definitive management [39]. Across the full dataset, reintervention rates ranged from 10% to 25%, consistent with the role of embolization as an adjunct or staging procedure within multimodal treatment frameworks.

Mortality following embolization was low, generally between 0% and 2%, with most deaths attributed to periprocedural hemorrhage or ischemic complications. In a series by Galaktionov et al., no treatment-related deaths occurred among 26 patients undergoing combined endovascular and surgical therapy [61].

Multimodality treatment: A total of 44 studies comprising 2,421 patients reported outcomes of multimodality therapy for cerebral AVMs. Although 52 studies described multimodal management strategies, only 44 provided extractable outcome data suitable for inclusion. The majority of protocols combined endovascular embolization with microsurgical resection, whereas selected cohorts incorporated adjunctive SRS or proton beam radiotherapy. Both staged and single-session hybrid workflows were represented; the latter enabled embolization and microsurgical resection within the same operative session under intraoperative digital subtraction angiography (DSA), allowing real-time confirmation of nidus eradication and minimizing interval hemorrhage. Functional outcomes across multimodality cohorts were generally favorable, with mRS 0-2 achieved in 72%-92% of patients. In the pediatric cohort by Winkler et al., mRS 0-2 was achieved in 81.5%, and 96.6% demonstrated neurological stability or improvement at last follow-up [119]. Similarly, Morgan et al. reported a total rate of mRS >2 of 10.4%, with poor outcomes strongly stratified by AVM grade: 5.4% for S-M <III, 16.8% for grade III, and 22% for grade >III lesions, highlighting the influence of lesion complexity on postoperative morbidity [141]. Long-term neurological recovery was consistently observed, and hybrid series frequently demonstrated favorable outcomes exceeding 90% at 6-12 months. In the study by Zeng et al., neurological function was improved or unchanged in 86.4% of multistage patients and 92.4% of hybrid patients at six months [130].

Obliteration rates for multimodality therapy were uniformly high, generally ranging from 90% to 100% across modern series. Zeng et al. reported complete occlusion in 96%-100% of cases following hybrid or staged embolization combined with microsurgery, with the hybrid arm achieving 100% angiographic cure on both early and mid-term imaging [130]. Single-session hybrid workflows consistently yielded the highest obliteration rates, reflecting the advantages of immediate intraoperative verification. Staged approaches demonstrated slightly lower rates attributable to small residual components in select high-grade or deep-seated lesions, whereas partial obliteration was uncommon and typically limited to diffuse nidus morphology or advanced S-M grades.

Complication rates varied with AVM complexity and institutional expertise. Hemorrhagic events occurred in approximately 6%-11%, consistent with postradiosurgical hemorrhage rates of 6.0%-8.2% reported by Chen et al. [104] and 10.6% in the Onyx plus Gamma Knife cohort described by Huo et al. [69]. Ischemic complications were reported in approximately 4%, largely attributable to embolic infarction of feeding vessels, as demonstrated by Morgan et al. [141]. Infection rates generally ranged from 10% to 13%, with Zeng et al. reporting intracranial infection in 12.9% and pulmonary infection in 4.6% of hybrid or multistage cases [130]. Neurological deficits occurred in 10%-25% of patients in the immediate postoperative period, with substantial improvement on follow-up; in the hybrid series by Zeng et al., neurological deficits decreased from 18.2% at discharge to 9.1% at three months and 5.3% at six months [130]. Residual AVM after intended curative therapy was uncommon, occurring in 2.3% of patients at three-month angiographic follow-up and 1.5% at six months [130]. Overall complication rates were lowest in hybrid series utilizing intraoperative DSA and optimized embolization strategies, which reduced ischemic injury and interval hemorrhage.

Mortality rates for multimodality therapy ranged from 1% to 4% across included studies. Most deaths were attributed to postoperative hemorrhage, cerebral edema, or herniation. The lowest mortality was reported in contemporary hybrid protocols, where immediate angiographic confirmation of cure minimized postoperative risk; Zeng et al. [130] documented 0% perioperative mortality, consistent with the 0%-2% mortality range observed in other modern cohorts [69,104].

Radiosurgery: In our review, 43 studies involving 13,162 patients reported outcomes of SRS for cerebral AVMs. These cohorts included both exclusive radiosurgery series and multimodal studies from which radiosurgery-specific data could be extracted. Across included studies, Gamma Knife and LINAC-based platforms predominated, with smaller subsets treated using CyberKnife (Accuracy Incorporated, Madison, WI) or proton beam systems. Most studies reported single-session SRS for appropriately selected lesions. Staged or repeat radiosurgery was employed for large-volume AVMs as well as for deep or residual lesions unsuitable for single-fraction treatment [116].

Functional outcomes following SRS were favorable in the majority of patients. Across studies that reported neurological status, 80%-95% of patients achieved an mRS score of 0-2 at last follow-up. Complete obliteration was achieved in 100% of patients treated with hypofractionated CyberKnife SRS in the series by Shah et al., with actuarial occlusion rates of 16.2%, 46.9%, and 81.1% at one, two, and three years, respectively [15]. Favorable outcomes were also reported by Yan et al., with 94.1% of patients achieving mRS 0-2, including 96.1% in the SRS-only cohort and 92.1% in the embolization+SRS cohort [110]. In contrast, functional deterioration was often associated with peritreatment hemorrhage. Sanchez-Mejia et al. reported neurological worsening in 24% of patients during the radiosurgical latency period, largely attributable to hemorrhagic events [32]. Long-term follow-up studies have reported favorable functional outcomes in the majority of patients after SRS. For example, Yan et al. reported that 94.1% of patients achieved mRS 0-2, including 96.1% in the SRS-only cohort and 92.1% in the embolization + SRS cohort [110].

Obliteration rates following radiosurgery varied widely, ranging from 22% to 100% across cohorts. The highest rates of complete angiographic cure were observed in small-volume or low-grade AVMs (S-M grades I-II), particularly those treated with single-session Gamma Knife radiosurgery. Zhu et al. demonstrated significantly higher obliteration rates in S-M I-II lesions compared with S-M III-IV after single-fraction SRS, while Yan et al. also highlighted the prognostic value of the modified radiosurgery-based AVM score, in which smaller nidus volume and lower scores are associated with improved treatment outcomes after SRS [13,110]. Conversely, larger, deep-seated, or eloquently located AVMs showed lower obliteration rates, typically 40%-70%, and frequently required repeat or volume-staged radiosurgical treatment. This pattern is exemplified by Shuto and Matsunaga, in whom AVMs averaging 24.8 mL treated with two- or three-stage Gamma Knife sessions achieved obliteration rates of 30.7% at three years and 58.2% at five years [113].

Complication rates ranged from 7% to 36%, depending on AVM characteristics, treatment dose, and follow-up duration. Hemorrhage during the latency period occurred in 0%-25% of patients; Abla et al. reported hemorrhagic events in up to 25% of high-grade AVM patients undergoing volume-staged SRS [31]. Radiation-induced necrosis was observed in 0%-32% of patients, although most cases were asymptomatic or transient. Lee et al. reported no necrosis in their cohort and noted that adverse radiation effects were rare and typically mild [116], whereas Choe et al. documented adverse radiation effects in 32% of patients, with only 6.6% being symptomatic and none resulting in permanent neurological deficits [115]. Neurological deficits across cohorts ranged from 0% to 96.1%, although contemporary series consistently reported persistent deficits in fewer than 15% of patients; Yan et al. demonstrated favorable long-term function in 92%-96% of treated patients [110]. Edema occurred in 0%-78.9% of patients and was generally transient; Shuto and Matsunaga observed T2 hyperintensity in 78.9% of patients, four of whom were symptomatic, although no permanent neurological deficits were reported [113]. Seizures were reported in 0%-84.6% of patients across series, yet clinically significant or new-onset seizures after SRS remained uncommon (≤15%); seizure outcomes following SRS were generally favorable. Peciu-Florianu et al. reported seizure freedom in 84.6% of patients with pretreatment epilepsy, while seizure exacerbation occurred in only 2.9% after Gamma Knife radiosurgery [66]. Reintervention for residual nidus was required in 0%-57% of patients. In the series by Esteves et al., four of seven patients with persistent AVM after radiosurgery (≈57%) underwent repeat SRS [76].

Overall complication rates were lowest in small, superficial AVMs and highest in large or eloquent lesions. Improvements in imaging-guided targeting, dose planning, and periprocedural management have significantly reduced symptomatic radionecrosis and neurological morbidity in modern series. Mortality following radiosurgery ranged from 0% to 12.5% across the included studies, with most deaths attributed to hemorrhage occurring during the latency period prior to complete obliteration, as illustrated by Abla et al. [31]. Contemporary series using modern Gamma Knife and LINAC systems reported far lower mortality rates, typically 0%-1%, reflecting improved patient selection and enhanced procedural safety. Koo et al. observed no treatment-related deaths among 154 patients treated with Gamma Knife radiosurgery [121].

Comparative Outcomes Across Modalities

**Figure 2:**
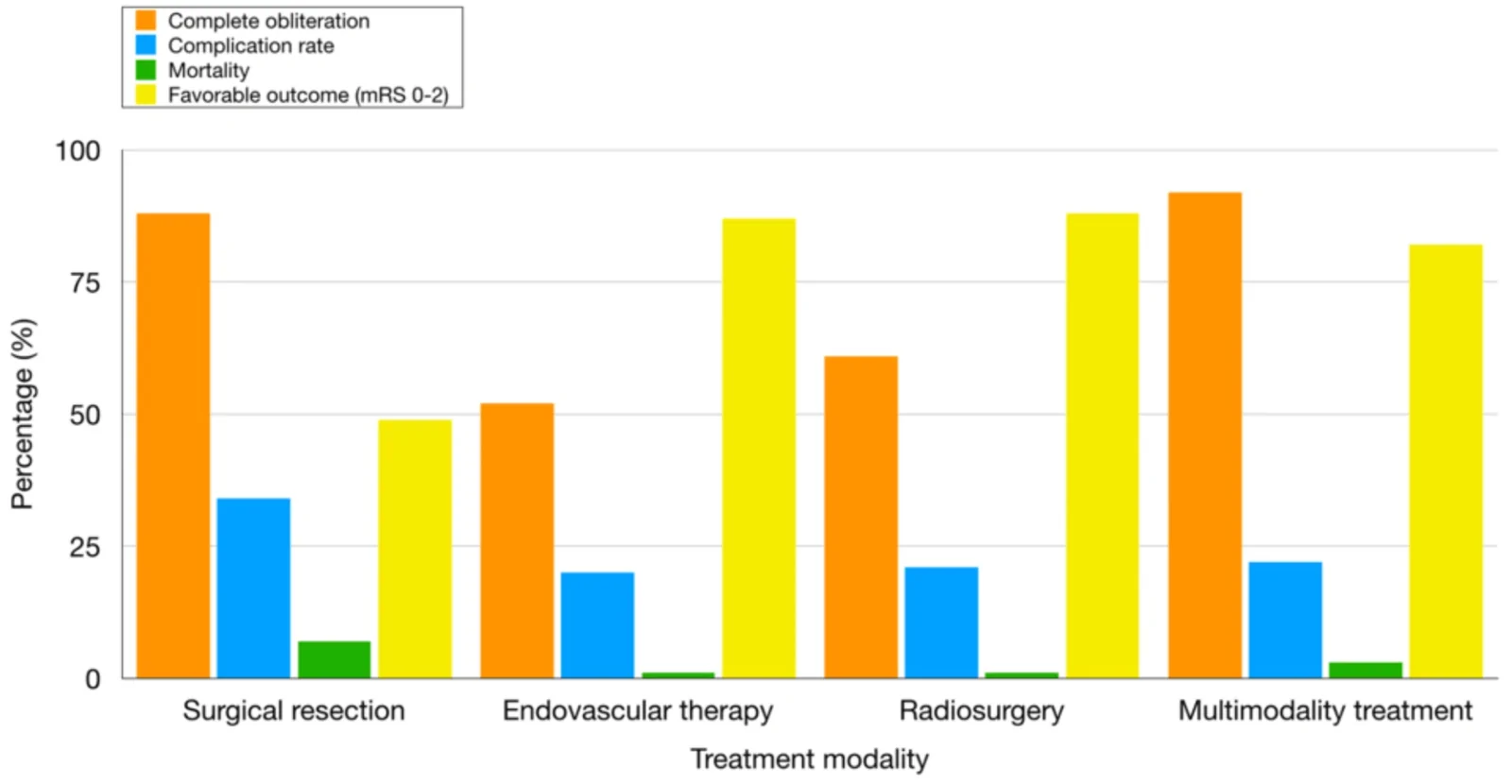
Summary of key outcomes across AVM treatment modalities Comparative distribution of major clinical outcomes across surgical resection, endovascular therapy, radiosurgery, and multimodality treatment for cerebral AVMs. Bars represent reported ranges of complete obliteration, complication rates, mortality, and favorable functional outcomes (mRS 0-2). Values represent heterogeneous descriptive ranges extracted from included studies and should not be interpreted as pooled comparative estimates or direct statistical comparisons between treatment modalities. Ranges only; values are presented for descriptive purposes and are not directly comparable across treatment modalities AVM: arteriovenous malformation; mRS: modified Rankin Scale Image credits: This image was synthesized by the authors Flora Alrumaih, Fadwa M. Albattah, Deem A. Almethen, and Abdulrahman M. Alharbi based on [9-141]

A comparative overview of obliteration, complication, mortality, and functional outcomes across all treatment modalities is presented in Figure 2 and summarized in Table 2. These visual representations allow direct comparison of the relative efficacy and safety profiles of microsurgery, endovascular therapy, SRS, and multimodal approaches. Together, the figure and table highlight the substantial variability in outcomes between modalities and illustrate the strengths and limitations of each treatment strategy based on the aggregated data from all included studies.

**Table 2:** Summary of outcomes across AVM treatment modalities Values represent ranges synthesized from the studies included in this systematic review and are based on extracted data. Reported rates summarize outcomes across eligible studies evaluating surgical resection, endovascular therapy, stereotactic radiosurgery, and multimodal treatment for cerebral AVMs. Data were derived from multiple sources including Zhu et al. [13], Shah et al. [15], Abla et al. [31], Sanchez-Mejia et al. [32], Peciu-Florianu et al. [66], Huo et al. [69], Esteves et al. [76], Chen et al. [104], Yan et al. [110], Shuto and Matsunaga [113], Choe et al. [115], Lee et al. [116], Winkler et al. [119], Koo et al. [121], Zeng et al. [130], Morgan et al. [141] AVM: arteriovenous malformation; mRS: modified Rankin Scale

Outcome	Surgical resection	Endovascular therapy	Radiosurgery	Multimodal
Complete obliteration (%)	78-100	5-92	22-100	90-100
Overall complication rate (%)	6.8-60	11.8-25	7-36	6-20
Mortality (%)	0-13.3	0-2	0-1	0-5
Favorable functional outcome (mRS 0-2, %)	4.9-92.9	71-95.5	80-95	72-92
follow-up duration (years)	0.25-10	0.25-11	1.25-15	0.17-20

Follow-Up and Comparative Trends Across Modalities

Based on pooled data from the included studies, substantial variability was observed in follow-up duration and outcome profiles across treatment modalities, highlighting important differences in both therapeutic objectives and durability of reported results.

Surgical series reported follow-up durations ranging from approximately 3 months to 10 years, enabling confirmation of angiographic cure and early postoperative neurological outcomes. Across these cohorts, microsurgical resection consistently achieved high rates of immediate obliteration (78%-100%) [34,38,61], although complication rates varied substantially depending on patient selection and lesion complexity, with higher rates reported in elderly or high-risk cohorts [44,132]. Mortality was generally low but reached up to 13.3% in selected high-risk populations, particularly elderly cohorts [44].

Endovascular series generally reported short- to mid-term follow-up durations (ranging from approximately three months to over 10 years, most commonly within three years), with outcomes primarily focused on procedural safety and nidus reduction rather than definitive cure. Across these studies, complete obliteration rates were highly variable (5%-92%), reflecting differences in AVM grade, embolic agents, and treatment intent [17,85]. Complication rates ranged from approximately 14% to 25%, encompassing hemorrhagic, ischemic, and procedure-related events [68,78]. These findings underscore the heterogeneous durability of endovascular outcomes and their predominant role as an adjunct within multimodal treatment strategies.

Radiosurgery cohorts reported the longest surveillance periods (approximately 15 months to 15 years), consistent with the delayed nature of nidus obliteration following SRS. Across these studies, obliteration rates ranged from 22% to 100% [13,15,110,113], contemporary mortality remained below 1% in modern series [121], and durable functional outcomes were frequently observed, with mRS 0-2 achieved in over 90% of patients in contemporary cohorts, with most studies reporting favorable outcomes in the majority of cases [110].

Multimodality strategies, including modern hybrid single-session protocols, demonstrated intermediate to long follow-up durations (two months to 20 years, most commonly 2-10 years). These approaches achieved high obliteration rates (90%-100%) [130], with complication rates reflecting a combination of hemorrhagic (6%-11%), ischemic (~4%), and infectious (10%-13%) events [69,104,130,141], and mortality generally ranging from 0% to 4% [69,104,130], reflecting the complementary integration of surgical, endovascular, and radiosurgical techniques to balance efficacy with risk.

Closing Summary

Microsurgical resection provides the most immediate and definitive obliteration, particularly for low-grade surgically accessible AVMs. Endovascular therapy demonstrates variable durability and is most commonly used as an adjunct within multimodality treatment strategies, whereas SRS achieves delayed but durable obliteration with favorable long-term outcomes. Multimodal approaches may facilitate treatment of complex lesions by combining the complementary advantages of individual modalities. Collectively, these findings highlight the importance of interpreting outcomes in the context of follow-up duration and therapeutic goals, immediate cure with surgery vs. delayed obliteration with radiosurgery, and underscore the need for individualized, lesion-specific treatment strategies tailored to angioarchitectural characteristics and patient factors [142].

Discussion

The management of cerebral AVMs remains a central challenge in neurosurgery because of their heterogeneous angioarchitecture, variable rupture risk, and differing responses to intervention. This review summarizes the reported outcomes of microsurgical resection, endovascular embolization, SRS, and multimodality strategies. Given the heterogeneity of patient characteristics, AVM features, and treatment selection across the included studies, these findings should be interpreted as descriptive rather than direct comparative evidence between treatment modalities.

Microsurgical Resection

Microsurgery consistently achieves the highest and most immediate obliteration rates, often exceeding 90% in contemporary multimodality cohorts [1]. Consensus recommendations also support microsurgical resection as the definitive curative option for small, superficially located AVMs when performed in experienced centers [2]. Additional single-institution series confirm high cure rates and acceptable morbidity for appropriately selected lesions [3]. These findings reinforce the role of surgery as first-line therapy for low-grade or surgically favorable AVMs.

Endovascular Therapy

Endovascular embolization demonstrated variable obliteration rates, reflecting differences in indication, technique, and nidus complexity across studies [39]. Modern approaches focus on tailored nidus reduction and hemodynamic stabilization as preparation for definitive microsurgical or radiosurgical therapy. Overall, current evidence supports embolization as a risk-modifying or adjunctive therapy rather than a reliable curative monotherapy.

Stereotactic Radiosurgery

SRS is commonly selected for small, deep-seated, or eloquently located AVMs that carry higher microsurgical risk, as reflected in contemporary management guidelines [142]. Earlier framework papers also highlight key considerations for noninvasive management of unruptured lesions [97]. In elderly patients, long-term neurological outcomes and mortality were similar across different management modalities, including SRS [38].

Multimodality Approaches

Combining treatment modalities can optimize outcomes for complex AVMs; series integrating endovascular embolization with microsurgical resection have demonstrated high obliteration rates with acceptable morbidity [19]. Modern targeted endovascular techniques also play a key role in reducing hemodynamic risk and facilitating safer definitive treatment [51]. Hybrid one-stage approaches, which integrate endovascular and microsurgical techniques, have demonstrated improved intraoperative control and high obliteration rates in contemporary multimodality cohorts [1,130].

Evidence Context: ARUBA and Subsequent Analyses

The ARUBA trial reported higher early stroke and death rates with intervention compared with conservative management for unruptured AVMs [143]. Its methodological rationale and design considerations were detailed in earlier publications [97]. However, limitations including low obliteration rates, short follow-up, and restrictive inclusion criteria limit generalizability. National data subsequently demonstrated that intervention rates for unruptured AVMs did not decline following ARUBA, suggesting persistent skepticism among neurovascular specialists regarding its applicability to real-world practice [43,90]. Collectively, these findings support conservative management for low-risk unruptured lesions while recognizing that selected patients with hemorrhagic presentation, progressive deficits, or surgically favorable anatomy may still benefit from active intervention.

Clinical Implications

Collectively, the evidence supports a risk-stratified approach. Microsurgery remains the definitive curative option for low-grade, accessible AVMs; radiosurgery offers durable results for deep or eloquent lesions with minimal invasiveness; and embolization serves primarily as an adjunct or targeted protective measure. Multimodal strategies, especially one-stage hybrid procedures, combine high obliteration rates with low morbidity and should be considered in complex or higher grade cases.

Limitations and Future Directions

Heterogeneity among included studies limits direct comparison, as AVM grade, outcome definitions, and follow-up durations varied considerably. Some series failed to differentiate single-modality from multimodal outcomes, and functional scales were inconsistently applied. Furthermore, this review synthesized data descriptively rather than meta-analytically. Additionally, the available evidence was predominantly derived from retrospective observational studies, with only four randomized controlled trials, limiting the overall strength of the conclusions. Furthermore, embolization outcomes were synthesized across studies with differing therapeutic intents (curative vs. adjunctive embolization), which may have contributed to the wide variability in reported obliteration rates. Future research should emphasize standardized reporting of mRS outcomes, time-to-obliteration, and hemorrhage during latency; prospective multicenter registries integrating hybrid-suite workflows; and long-term studies that capture late complications and durability of cure.

## Conclusions

The present synthesis underscores that the management of cerebral arteriovenous malformations is best guided by precision rather than preference. Across contemporary literature, durable cure and functional preservation depend more on appropriate selection and sequencing than on the superiority of any single technique. Microsurgery delivers immediate cure where anatomy permits, radiosurgery offers durable results for deep or eloquent lesions, and embolization enhances the safety and success of both when applied judiciously.

The collective evidence emphasizes that progress in AVM care lies in integration-of modalities, expertise, and technology. Future efforts should focus on prospective, data-driven frameworks that incorporate patient-specific risk modeling, hybrid neurovascular suites, and long-term functional endpoints to redefine what constitutes true cure and quality of life in AVM treatment.
